# Analysis of muscle mass by computed tomography in patients with head and neck cancer: a prospective study

**DOI:** 10.1590/0100-3984.2023.0037

**Published:** 2023

**Authors:** Thais Manfrinato Miola, Almir Galvão Vieira Bitencourt, Juliana de Oliveira Souza, Luiz Paulo Kowalski, João Gonçalves Filho

**Affiliations:** 1 A.C.Camargo Cancer Center, São Paulo, SP, Brazil

**Keywords:** Head and neck neoplasms, Body composition, Tomography, X-ray computed, Nutritional assessment, Preoperative period., Neoplasias de cabeça e pescoço, Composição corporal, Tomografia computadorizada por raios X, Avaliação nutricional, Período pré-operatório.

## Abstract

**Objective:**

To evaluate the preoperative muscle mass of patients with head and neck cancer (HNC) with computed tomography (CT), comparing the results obtained through analysis of cross-sectional areas at the level of the third lumbar vertebra (L3) with those obtained through analysis of cross-sectional areas at the levels of the third cervical and fourth thoracic vertebrae (C3 and T4, respectively).

**Materials and Methods:**

A total of 63 patients with HNC were evaluated preoperatively. Using CT, we assessed muscle mass at L3, as well as at C3 and T4.

**Results:**

Most (73.0%) of the patients had low muscle mass at L3, whereas 50.8% had a normal body mass index. The cross-sectional area at L3 correlated strongly with those at C3 and T4 (r = 0.831 and r = 0.763, respectively; *p* < 0.001 for both). In addition, the muscle mass index at L3 correlated strongly with those at C3 and T4 (r = 0.781 and r = 0.715, respectively; *p* < 0.001 for both).

**Conclusion:**

Low muscle mass appears to be highly prevalent in patients with HNC. Measurements at C3 and T4 could represent alternative means of assessing muscle mass in such patients.

## Abstract (pt)

**Objetivo:**

Avaliar e comparar a massa muscular de pacientes com câncer de cabeça e pescoço (CCP) durante o período pré-operatório com tomografia computadorizada (TC), por meio da análise das áreas transversais no nível da terceira vértebra lombar (L3) em comparação com níveis cervical (C3) e torácico (T4).

**Materiais e Métodos:**

Sessenta e três pacientes com CCP foram avaliados no pré-operatório. A TC foi utilizada para avaliar a massa muscular de L3, assim como as secções transversais de C3 e T4.

**Resultados:**

A maioria dos pacientes (73,0%) tinha baixa massa muscular analisada pela TC de L3, mas índice de massa corpórea normal (50,8%). Também foi observada forte correlação entre as áreas e o índice de massa muscular (IMM) de L3 com C3 (área: r = 0,831, *p* < 0,001; IMM: r = 0,781, *p* < 0,001) e T4 (área: r = 0,763, *p* < 0,001; IMM: r = 0,715, *p* < 0,001).

**Conclusão:**

A baixa massa muscular é altamente prevalente em pacientes com CCP. As análises de TC em C3 e T4 podem representar opção para avaliar a massa muscular em pacientes com CCP.

## INTRODUCTION

Head and neck cancer (HNC) is the seventh leading type of cancer worldwide, accounting for approximately 890,000 newly diagnosed cases and 450,000 deaths in 2018^**(^1^)**^. Most HNCs are diagnosed at an advanced stage and require multimodal treatment with a combination of surgery, radiotherapy, and chemotherapy. As a result, there can be significant functional sequelae (affecting chewing and swallowing), weight loss, and changes in body composition^**(^2^)**^.

Patients with HNC are at a high nutritional risk, and weight loss is common before, during, and after treatment^**(^3^)**^. At diagnosis, approximately 60% of patients are malnourished^**(^4^,^5^)**^. Subsequent weight loss during treatment can affect up to 80% of patients, with 70% of the weight lost being fat-free mass^**(^5^)**^.

Malnutrition associated with HNC has been found to be multifactorial. It is related to difficulty in eating caused by obstructive dysphagia, metabolic changes, and treatment morbidity. Malnutrition has also been associated with increased postoperative complications, increased risk of infection, reduced wound healing, increased length of hospital stay, higher treatment cost, decreased tolerance to treatment, worse quality of life, and higher mortality^**(^4^,^6^)**^. Therefore, nutritional assessment is essential for HNC patients, with the objective of diagnosing nutritional status and determining the appropriate nutritional therapy aimed at improving clinical outcomes^**(^7^,^8^)**^.

Body composition analysis is an integral part of a nutritional status assessment. Anthropometry, bioelectrical impedance analysis, dual energy X-ray absorptiometry, and computed tomography (CT) are the methods indicated^**(^9^,^10^)**^. Currently, CT is used in assessments of body composition, the analysis of cross-sectional areas at the level of the third lumbar vertebra (L3) allowing quantification of subcutaneous and visceral adipose tissue of the psoas, paraspinal, and abdominal muscles, and thus muscle mass, to be quantified. Such analysis shows a strong correlation with whole-body fat and muscle mass. Consequently, it has become the gold standard for this type of evaluation, especially in cancer patients^**(^8^,^11^-^13^)**^. However, CT of the abdomen is not routinely performed for the staging of patients with HNC. More often, CT scans of the neck and chest are acquired. It has been proposed that muscle mass should be assessed at the level of the third cervical vertebra (C3) or the fourth thoracic vertebra (T4), rather than at the L3 level^**(^10^,^13^,^14^)**^. However, that assessment has yet to be validated.

The aim of the present study was to use CT to analyze the muscle mass of patients with HNC, correlating the findings obtained at L3 with those obtained at C3 and T4.

## MATERIALS AND METHODS

This was a prospective cohort study conducted from August 2017 to November 2020. The study was approved by the Research Ethics Committee of A.C.Camargo Cancer Center (Reference no. 2362/17), and all participating patients gave written informed consent.

We included patients with HNC that were admitted for treatment in the Department of Head and Neck Surgery and Otolaryngology. The inclusion criteria were being ≥ 18 years of age and having been diagnosed with squamous cell carcinoma of the upper aerodigestive tract. Patients who had previously undergone cancer treatment were excluded, as were those with a history of surgery in the head and neck region, those with synchronous tumors that would have required different, simultaneous treatments, and those who had not undergone CT of the abdomen. Thus, of the 423 patients who underwent surgery for the treatment of squamous cell carcinoma of the upper aerodigestive tract during the study period, 360 were excluded: 177 because they did not undergo CT of the abdomen, 105 because they had a history of surgery in the head and neck region, and 78 because they had previously undergone cancer treatment. Therefore, the final sample comprised 63 patients. Because of the availability of CT images, it was possible to correlate the cross-sectional areas at L3 with those at C3 in only 52 patients and with those at T4 in only 60.

### Nutritional assessment and muscle mass

Muscle mass was assessed on CT by an experienced radiologist during the preoperative period in coordination with examinations for cancer staging. Anthropometric data, such as weight, height, and body mass index (BMI), were also evaluated. The BMI reference values used for patients up to 59 years of age were those established by the World Health Organization^**(^15^)**^; for patients ≥ 60 years of age, the Pan American Health Organization reference values were used^**(^16^)**^.

**Figure 1:**
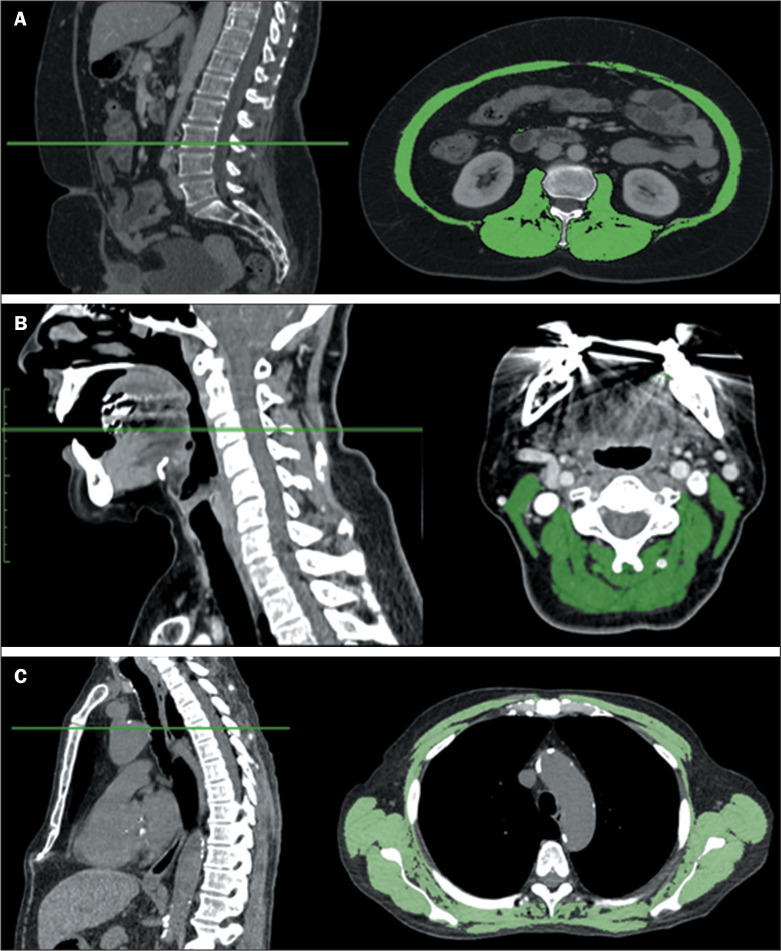
A: Example of skeletal muscle area measurement at the L3 level. B: Paravertebral and sternocleidomastoid muscle areas measurements at the C3 level. C: Example of skeletal muscle area measurement at the lower part of the T4 level.

Axial CT slices acquired at L3 were analyzed with OsiriX software, version MD ANVISA (Pixmeo SARL, Bernex, Switzerland) to determine the muscle mass (Figure 1A). To measure the area of muscle mass (skeletal musculature, including the psoas, paravertebral, and abdominal wall muscles), we used a semi-automatic method with manual correction, when necessary. The skeletal musculature was identified by its density (from -29 to +150 HU). To calculate the muscle mass index (MMI), muscle mass area was corrected for height (muscle mass in cm^2^/height in m^2^). To classify muscle mass depletion, we used MMI cutoff values of < 55 cm^2^/m^2^ for men and < 39 cm^2^/m^2^ for women^**(^17^)**^. For comparison, muscle mass was also evaluated at the C3 and T4 levels. The musculature of the cervical region, including the paravertebral and sternocleidomastoid muscles^**(^18^)**^, was analyzed in an axial CT slice acquired at the C3 level (Figure 1B). The musculature of the thoracic region, including the pectoralis, intercostalis, paraspinal, serratus, and latissimus muscles^**(^19^)**^, was analyzed in an axial CT slice acquired at the T4 level (Figure 1C).

### Statistical analysis

Statistical analyses were performed by using the IBM SPSS Statistics software package, version 22.0 (IBM Corp., Armonk, NY, USA). Continuous variables are presented as median or mean and standard deviation, for non-normal and normally distributed data, respectively. Normality was tested by applying the Kolmogorov-Smirnov test. Ordinal or nominal variables are presented in absolute number values and percentages; the percentages of the total were calculated for the categorical variables. Pearson’s or Spearman’s correlation coefficient was used in order to determine how strongly the skeletal muscle area and MMI measured at L3 correlated with those same parameters measured at C3 and T4. Linear regression was performed to assess the associations among and influence of muscle mass areas and indices at L3, C3, and T4. Values of *p* < 0.05 were considered statistically significant.

## RESULTS

Of the 63 patients included in this study, the majority (77.8%) were men. The median age was 58 years (range, 26-93 years). Twenty-eight patients (44.4%) reported smoking, and 19 (30.2%) reported consuming alcoholic beverages. Most of the patients (71.4%) had a tumor in the oral cavity, and the majority (76.2%) were diagnosed at an advanced stage. The median body weight was 68 kg (range, 46.2-125 kg). More than half of the patients (50.8%) had a normal BMI (Table 1).

**Table 1:** Clinical and anthropometric characteristics of patients with HNC.

Variable	(N = 63)
Sex, n (%)	
Male	49 (77.8)
Female	14 (22.2)
Age (years)	
Median (range)	58 (26-93)
Mean ± SD	57.8 ± 12.7
Tumor site, n (%)	
Oral cavity	45 (71.4)
Oropharynx	9 (14.3)
Larynx	8 (12.7)
Maxillary sinus	1 (1.6)
Clinical stage, n (%)	
I-II	15 (23.8)
III-IV	48 (76.2)
Weight (kg)	
Median (range)	68.0 (46.2-125.0)
Mean ± SD	69.8 ± 15.5
Height (m)	
Median (range)	1.69 (1.51-1.90)
Mean ± SD	1.69 ± 0.08
BMI (kg/m^2^)	
Median (range)	23.8 (16-38)
Mean ± SD	24.2 ± 4.4
BMI classification, n (%)	
Underweight	12 (19)
Normal weight	32 (50.8)
Overweight/obese	19 (30.2)

SD, standard deviation.

Muscle mass at the L3 level, as determined by CT, showed wide variation between patients, with a median value of 130.7 cm^2^. However, MMI was classified as below normal in 73% of the patients. There was no statistically significant association between low MMI and tumor stage (*p* = 0.523). At the T4 and C3 levels, the median muscle mass was 200.3 cm^2^ and 40.3 cm^2^, respectively (Table 2).

**Table 2:** Assessment of muscle mass by CT in patients with HNC.

Variable	N (%)
Muscle mass area (cm^2^) at L3	
Median (range)	130.7 (80.1-242.4)
Mean ± SD	133.4 ± 30.8
MMI (cm^2^/m^2^) at L3	
Median (range)	46.6 (27.7-78.2)
Mean ± SD	46.3 ± 9.2
Classification of MMI at L3	
Adequate	17 (27)
Inadequate	46 (73)
Muscle mass area (cm^2^) at T4	
Median (range)	200.3 (110.5-328.1)
Mean ± SD	197.5 ± 47.7
MMI (cm^2^/m^2^) at T4	
Median (range)	67.5 (43.7-105.9)
Mean ± SD	68.7 ± 15.1
Muscle mass area (cm^2^) at C3	
Median (range)	40.3 (25.1-62.6)
Mean ± SD	42.1 ± 9.4
MMI (cm^2^/m^2^) at C3	
Median (range)	14.0 (9.6-20.1)
Mean ± SD	14.5 ± 2.8

SD, standard deviation.

**Figure 2:**
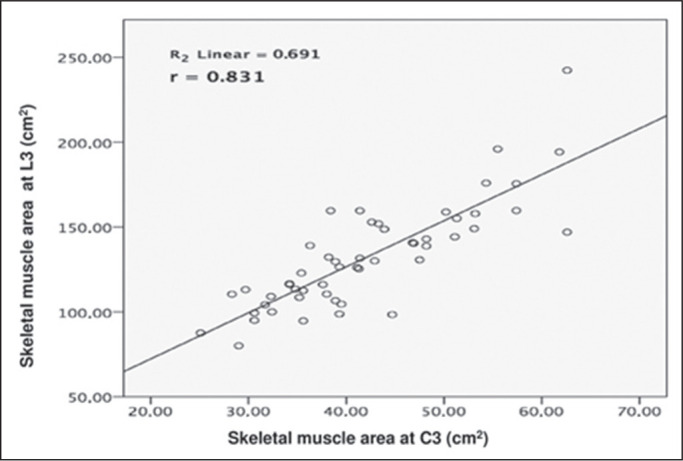
Correlation between L3 and C3 muscle mass area (cm^2^). A positive relationship is presented (r = 0.831; *p* < 0.001).

**Figure 3:**
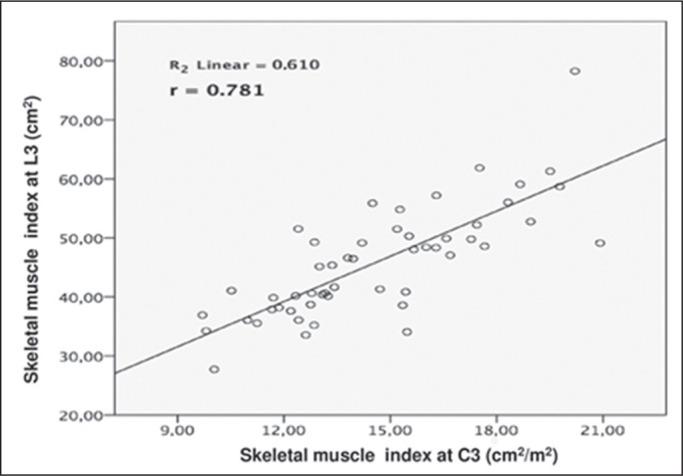
Correlation between L3 and C3 MMI. A positive correlation was observed (r = 0.781; *p* < 0.001).

**Figure 4:**
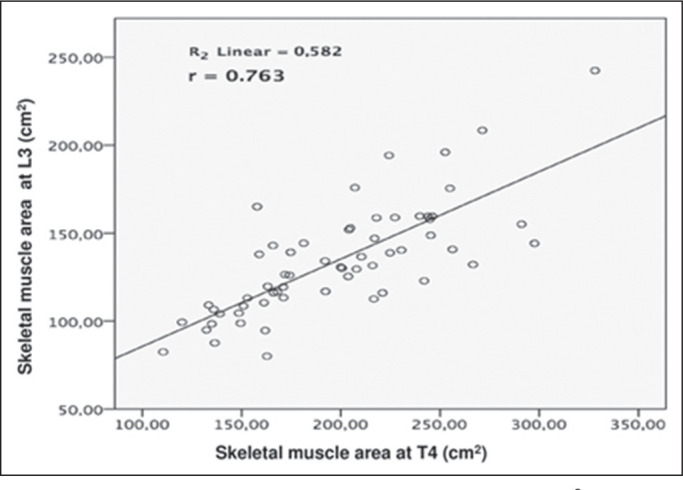
Correlation between L3 and T4 muscle mass area (cm^2^). A positive relationship is noted (r = 0.763; *p* < 0.001).

**Figure 5:**
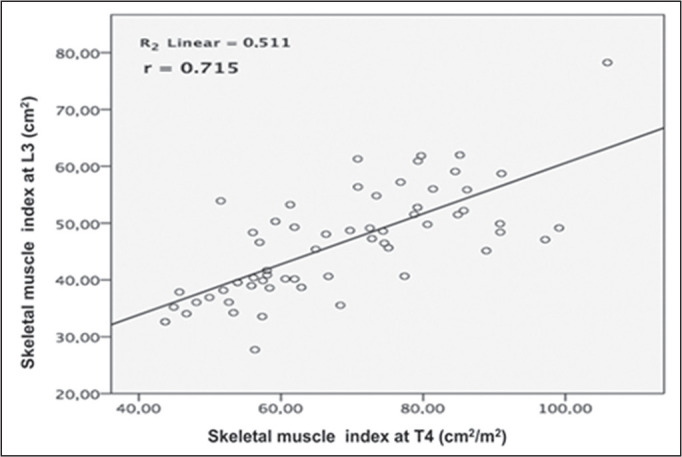
Correlation between L3 and T4 MMI. A positive correlation was observed (r = 0.511; *p* < 0.001).

Muscle mass area was assessed at the T4 level in 60 patients and at the C3 level in 52 patients. An analysis of the muscle mass areas at L3 and C3 showed a strong correlation between the two (r = 0.831; *p* < 0.001), as illustrated in Figure 2. There was also a strong correlation between the MMI calculated at L3 and that calculated at C3 (r = 0.781; *p* < 0.001), as shown in Figure 3. In addition, when the values obtained at L3 and T4 were compared, a strong correlation was observed between the muscle mass areas (r = 0.763; *p* < 0.001) (Figure 4), and the MMIs (r = 0.715; *p* < 0.001) (Figure 5).

## DISCUSSION

In most cases, HNC is diagnosed at an advanced stage, causing significant morbidity, including weight loss and malnutrition. Approximately 60% of HNC patients are malnourished at the time of diagnosis^**(^5^)**^. In the present study, approximately 20% of the patients were classified as malnourished on the basis of their BMI. In addition, in most of the patients, CT revealed low muscle mass prior to the start of treatment.

The assessment of nutritional status and the diagnosis of malnutrition are important steps to be taken before cancer treatment is initiated, because that knowledge can define care and its application can reduce treatment morbidity, thus improving outcomes. Various methods are employed to assess the nutritional status of cancer patients^**(^8^,^9^,^17^)**^. Among those, a traditional anthropometry measure, the BMI, is widely used. However, the BMI does not differentiate between fat mass and muscle mass. Therefore, it should not be considered in isolation, because it could delay the diagnosis of malnutrition. As previously mentioned, most of the patients in our sample (50.8%) had a normal BMI. However, CT showed low muscle mass in 73% of the sample. Nishikawa et al.^**(^20^)**^ obtained similar results for BMI and MMI. Those authors found that, when muscle mass at L3 was analyzed by CT in patients diagnosed clinically and surgically with HNC, it was categorized as low in 46.0% of the patients, whereas 49.4% of the patients had a normal BMI.

In recent decades, the quantification of muscle mass area by CT has been regarded as the gold standard in the assessment of body composition in cancer patients^**(^21^)**^. As such, it is now part of the diagnostic workup and is used to determine staging, as well as for monitoring during follow-up^**(^22^,^23^)**^. However, it is not applied in clinical practice for assessments of body composition. In the present study, the muscle mass area observed in CT slices acquired at C3 and T4 showed a significant correlation with that observed in CT slices acquired at L3. Therefore, muscle mass could be analyzed in patients with HNC who do not undergo abdominal CT.

In the present study, 73% of the patients had a below-average MMI. Similarly, Achim et al.^**(^24^)**^ observed that 77% of patients undergoing total laryngectomy had low muscle mass, which they found to correlate significantly with the morbidity related to the surgical treatment.

The assessment of body composition through analysis of muscle mass at the C3 level is an economical alternative in patients with HNC who have not been treated previously and do not undergo CT of the abdomen. In the present study, we detected a strong, significant correlation between the muscle mass area observed at L3 and that observed at C3, as well as between the MMI observed at L3 and that observed at C3. Swartz et al.^**(^18^)**^ observed a strong correlation between the C3 and L3 areas in 103 patients, 52 of whom had HNC. In a study of 159 patients with HNC, Ufuk et al.^**(^14^)**^ compared the MMI at C3 with that at L3 and detected a strong correlation between the two. Finally, a systematic review conducted by Almada-Correia et al.^**(^5^)**^ showed that muscle mass measured at C3 correlates significantly with that measured at L3, making its measurement at C3 a reliable method to be indicated for patients with HNC. However, Yoon et al.^**(^25^)**^ did not observe such agreement in their study of 165 HNC patients and therefore stated that measuring muscle mass at C3 is not recommended as a diagnostic modality in patients with sarcopenia. The concern is that measurements at C3 might not be useful for follow-up evaluations in many patients because of surgical resection of the sternomastoid muscle and the local effects of radiation.

Muscle mass at the T4 level has been studied in cancer patients, although mainly in those with lung cancer^**(^19^,^22^,^23^)**^. In the current study, when the muscle mass area and the MMI were analyzed at the T4 and L3 levels, a strong correlation between the two levels was observed for both parameters. Using those same two parameters, Wysham et al.^**(^26^)**^ observed a moderate correlation in patients with lung cancer. In another study of patients with lung cancer, Grønberg et al.^**(^23^)**^ also detected a moderate correlation between the muscle mass areas at T4 and L3. A CT slice acquired at T4 can be more appropriate for follow-up, because thoracic CT is routinely performed and the region is not affected by the locoregional treatments.

Our study has some limitations, not the least of which is the small sample size, That is attributable to various causes, including the fact that abdominal CT images were not available for many patients and the occurrence of the coronavirus disease 2019 pandemic, which limited the number of patients eligible for inclusion in the study. In addition, we analyzed muscle mass only in the preoperative period. We opted not to consider values obtained at the beginning of treatment for patients who underwent neoadjuvant or induction chemotherapy, because of the risk of bias, given that antineoplastic therapy can reduce muscle mass through the metabolization of certain drugs or the nutritional impact of drug toxicity. Furthermore, the lack of a control group of non-comparison of muscle mass analysis with healthy individuals precluded the establishment of cutoff values for the indices measured at C3 and T4. There is therefore a need for comparative studies in order to define those cutoffs.

## CONCLUSION

Assessment of the nutritional status of cancer patients is essential to optimize their nutritional care. Consequently, it is important to choose and validate the most accurate tools. By monitoring the nutritional status of patients, specific strategies can be implemented to improve treatment outcomes. The results of the present study indicate that there is a high prevalence of low muscle mass among patients with HNC and that muscle mass at L3 correlates significantly with muscle mass at C3 and T4. Although more studies are needed in order to confirm that correlation, CT measurements at C3 (prior to any locoregional treatment) and T4 could represent alternatives to assess muscle mass in HNC patients, especially when images acquired at L3 are not available.
